# Prevalence of Vertical Root Fractures in a University Endodontics Program versus a Private Endodontics Office

**DOI:** 10.1155/2023/2098629

**Published:** 2023-12-19

**Authors:** Berit Ek, Stefan Zweig, Ramon G. Roges, Yaara Berdan, Rafael A. Roges, Ayman Abulhamael, Abrar Kutbi, Alaa W. Alqutub, Osama S. Alothmani, Amna Y. Siddiqui

**Affiliations:** ^1^Herman Ostrow School of Dentistry, University of Southern California, Los Angeles, CA, USA; ^2^Department of Endodontics, Faculty of Dentistry, King Abdulaziz University, Jeddah, Saudi Arabia; ^3^University Dental Hospital, King Abdulaziz University, Jeddah, Saudi Arabia; ^4^Department of Oral and Maxillofacial Surgery and Diagnostic Sciences, Umm Al-Qura University, Makkah, Saudi Arabia

## Abstract

**Introduction:**

Vertical root fractures (VRFs) typically have a poor prognosis with an extraction or occasionally root amputation as the preferred treatment. VRFs have been considered an epidemic, motivating changes in the access openings, as well as in the preparation and disinfection protocols of endodontic treatment. Hence, we aimed to evaluate the prevalence of VRFs by tracking cases in both a University Endodontic Program (UEP) and a private endodontics practice (PP). We also evaluated changes in prevalence during COVID-19 along with the alterations in the frequency of cases diagnosed by clinical and radiographic signs which were later confirmed by direct visualization compared to those in which the suspicion was based on clinical and radiographic signs alone.

**Methods and Materials:**

This retrospective study looked at the prevalence of VRF in patient records at UEP and a PP. Data for the pre-COVID-19 and COVID-19 time periods were extracted from patient records and referral letters then compared. Data for suspected and confirmed prevalence were compared.

**Results:**

The UEP group included 21,156 patients while the PP group comprised 7,209 patients. The prevalence of VRFs in the former cohort was 1.80%, while 2.62% of the latter cohort exhibited VRFs with a combined total of 2.01%. The combined total prevalence of VRFs pre-COVID-19 was 1.72%, increasing from 2.1% to 3.82% during COVID-19 (*p* < 0.0001). The same applied to suspected cases for both clinical settings. The increase in confirmed cases between the two periods was statistically significant for the UEP group (*p*=0.0202) but it was insignificant for the PP group (*p*=0.0721).

**Conclusion:**

The combined prevalence for VRFs was 2.01% for all years denying the claim that VRF is a pandemic phenomenon. COVID-19 period saw almost a double increase in the prevalence of VRF compared to pre-COVID-19 era. This was consequently associated with a significant increase in the number of suspected VRF cases.

## 1. Introduction

Longitudinal tooth fractures are clinical conditions that are difficult to diagnose and manage. They are classified into five categories including craze lines, fractured cusps, cracked teeth, split teeth, and vertical root fractures (VRFs) [1]. Complete VRF is defined as a fracture that extends from one proximal aspect to the opposite side of the root while an incomplete VRF involves only one side of the root [2]. VRFs are catastrophic incidences and around 7% of failed nonsurgical treatment cases were due to VRF [3]. Around 11% of root-filled teeth were extracted due to VRF [4].

The etiology of VRF is not fully understood but they are believed to be related to postplacement and condensation forces during root canal filling [1, 5–11]. VRFs usually occur in endodontically treated teeth [12, 13] especially molars [2, 13]. In theory, microcracks can be generated during endodontic procedures or postplacement and may grow over time due to cyclic loading of the tooth [14, 15]. According to Moule and Kahler [16], the average time between root filling and the appearance of a VRF has been estimated to be between 39 and 52.5 months with a range variance of 3 days to 14 years. There have also been case reports of instances where VRFs occur in nonendodontically treated teeth, particularly in the elderly Chinese population and in first molars [1, 6, 17–20].

Although there are certain signs that typically appear with VRFs, they are difficult to diagnose with only one-third of general practitioners correctly identifying them [21]. These signs include the history of endodontic treatment, fracture running faciolingually, postplacement, swelling, pain upon biting, presence of indirect restoration, and/or sinus tract in the attached gingiva [2, 6, 13]. About 90% of VRF cases had dehiscence in the buccal plate [22]. Therefore, a deep narrow isolated pocket is a strong indicator for a VRF. Percussion and palpation tests are usually not a strong indicator. However, many of these signs and symptoms are the same for a primary endodontic disease with drainage through the periodontal ligament. These signs can only lead one to suspect that there is a VRF [22]. A systematic review reported that the most common clinical signs of VRF were deep narrow pocket and coronally located sinus tract. The halo appearance was the most common radiographic sign. However, scientific evidence supporting these clinical and radiographic signs was lacking [23].

VRFs are difficult to diagnose especially when the segments are not separated. Presence of root filling negatively impacted the radiographic detection of VRF [24, 25]. Bone loss patterns seen in radiographic images may be indicative of a VRF, and occasionally it is possible to see the fracture on a periapical radiograph and a cone beam computed tomography (CBCT) [2, 6, 13, 26]. For this to be depicted on a periapical radiograph, the separation would be wide enough to show that the root is split or the angle of the beam must line up exactly with the fracture. According to the Nyquist theorem, for a CBCT to show a VRF, the separation between the two segments of the fracture must be double the voxel size for the fracture to be discernable [27]. Due to the variety of presentations and overlap with “endo-perio” conditions, direct visualization of the VRF remains the gold standard in its diagnosis. It is important to understand VRFs compared to other longitudinal fractures to diagnose and treat them properly [22].

The primary aim of this study was to evaluate the prevalence of VRFs by tracking cases in both a postgraduate program and a private endodontics practice (PP). The second aim of the study was to evaluate the change in prevalence during COVID-19. The third aim of the study was to evaluate how many of these cases were diagnosed by clinical and radiographic signs which were later confirmed by direct visualization compared to those in which the suspicion was based on clinical and radiographic signs alone.

## 2. Materials and Methods

This retrospective study was conducted within one University Endodontics Program (UEP) and one Private Endodontics Office (PP). Appropriate ethical approval (IRB number HS-22-00067) was obtained to review the university Axium records. A search through the electronic records database was done to identify all notes related to VRFs.

The UEP subset used the following years: January 2011 to March 2022. Pre-COVID-19 years consisted of records from January 2011 to December 2019. COVID-19 years consisted of January 2020 to March 2022. For the PP subset, the dates started in 2006 and ended at the same time as the UEP subset in March 2022.

### 2.1. University Endodontics Program (UEP)

Within the database of Axium notes from UEP, only the advanced endodontics department notes were searched for that period of time. A report on the total number of notes written was run, as well as the total number of patients seen. The search terms occurred in the following order: “vertical root fracture,” “VRF,” “fracture,” “longitudinal root fracture,” “longitudinal fracture,” “longitudinal crack,” “root fracture,” and “vertical.” After each term was searched, that note was keyworded accordingly. Once it was keyworded, it was filtered out in order to examine all remaining notes. This allowed for unsearched terms containing “vertical” that should have been written as “vertical root fracture” to be included in the search. One example of that came up was “vertical root crack.” There were also occasions where parts of “vertical root fracture” were misspelled that did not allow them to be searched with “vertical root fracture.” Thus, it was necessary to search other terms such as “fracture” or “vertical” and eliminate from there. At the same time, other phrases that included the word “vertical” could be clearly excluded from the study such as “warm vertical” and “vertical release.”

Once the data were searched, further classification was done to determine whether the VRF was suspected, confirmed radiographically, visually, surgically, or confirmed with no fracture present, misdiagnosed, part of an informed consent, or was a duplicate, indicating that it was the same patient and same tooth. For example, a consult record and then an exploratory surgery record on another day. Duplicates were eliminated from the total count.

### 2.2. Private Endodontics Office (PP)

For the PP, the treatment letters were used for the search which was equivalent to the number of patients seen. A total of 7,209 treatment letters were searched for a set period of time. These treatment letters were searched for the keywords “vertical” and “VRF.” All letters with these keywords were examined for their relation to VRFs. Only the number of letters related to VRFs was included into the count of this study. The years examined were for the total overall in 2006–2022, pre-COVID-19 during 2006–2019, and during COVID-19 in 2020–2022.

Once the data were searched, further classification was done to determine whether the VRF was suspected, confirmed radiographically, visually, surgically, or confirmed with no fracture present.

### 2.3. Categories

(1) *Suspected* indicated that the provider suspected the tooth had a VRF, but the patient decided to proceed with endodontic treatment, extract the tooth, or monitor it.
(2) *Confirmed positive (+)* indicated that the provider visually, radiographically, or surgically confirmed that there was a VRF present.
  - *Confirmed radiographically* (*PP only*) indicated that there was a clear fracture seen on the digital periapical radiograph as specified in the treatment letter. For the UEP group, radiographs were not available. Only a few treatment records specifically mentioned that the VRF was clearly seen on the digital periapical radiographs.
  - *Confirmed visually* indicated that in areas of deep periodontal pocketing, the gingiva was reflected with an instrument such as the Glick, and the surface was stained with methylene blue dye to determine if a crack or fracture existed.
  - *Confirmed surgically* indicated that the patient was anesthetized, and a flap was laid to visualize the fracture with methylene blue dye or transillumination. It also may indicate that endodontic surgery was performed to eliminate the fractured segment.
(3) *Confirmed negative (−)* indicated that cases were suspected but not present for VRF when looked for surgically or visually.

### 2.4. Statistical Analysis

Once all the data were keyworded for both the UEP and the PP, the prevalence of VRF was calculated. A one-tailed *z*-test was performed with a confidence level of 95%.

## 3. Results

### 3.1. Overall Prevalence

Total UEP records reviewed was 60,733 as opposed to 7,209 patients reports at PP totaling 67,982. The total number of patients seen for the UEP was 21,156 and 7,209 reports for the PP with a combined total of 28,365 patients or reports. Regarding the overall prevalence; UEP had a prevalence rate of 1.80% and the PP had a prevalence rate of 2.62%, for a combined prevalence rate of 2.01% (Table 1).

### 3.2. Pre-COVID-19 vs. COVID-19 Prevalence

The total prevalence of VRF based on the number of patients was 1.72% pre-COVID-19, with an increase to 3.82% during COVID-19. This 2.1% increase was statistically significant (*p* < 0.0001).

### 3.3. University Endodontics Program: Suspected vs. Confirmed

For the UEP set, the years for pre-COVID-19 ended in December 2019, while the COVID-19 subset started in January 2020 and ended in March 2 years later. Within the UEP database, the prevalence of suspected VRFs was 0.93% and confirmed with VRF present at 0.69%. Regarding the COVID-19 period subset, there was an increase in the suspected prevalence from 0.78% to 1.91%, which was statistically significant (*p* < 0.0001). The cases that were confirmed to have a VRF saw an increase from 0.65% to 0.99% which was statistically significant (*p*=0.0202, Table 2).

### 3.4. Private Endodontics Office (PP)

In general, each treatment letter was associated with one tooth. There were a few occasions where the treatment letter had multiple teeth involved. For the PP subset, the years included for COVID-19 started in January 2020 and went to March 2022. The total records that were reviewed were 7,209 for all years and 1,018 for the COVID-19 years. The prevalence of suspected VRF was 1.87% and confirmed with VRF present was 0.72%. Regarding the COVID-19 period subset, there was an increase in the suspected prevalence from 1.62% to 3.44% which was statistically significant. The cases that were confirmed to have a VRF saw an increase from 0.66% to 1.08% which was not statistically significant (Table 3).

Based on the PP, there was a statistically significant difference in the pre-COVID-19 and during COVID-19 periods for the suspected prevalence (*p* < 0.0001) and when suspected and confirmed prevalence (positive and negative) were combined (*p* < 0.0001). However, when examining the actual confirmed positive (*p*=0.0708) and confirmed negative (*p*=0.0721) cases, the difference was not statistically significant (Table 3).

### 3.5. University Endodontics Program and Private Endodontics Practice Combined: Suspected vs. Confirmed

When combining the sources of data, the prevalence for all years was 2.01%. When looking at the change over the years, there was an increase from 1.72% pre-COVID-19 to 3.82% during COVID-19 which was statistically significant (*p* < 0.001). When looking at the suspected prevalence, there was an increase from 0.99% pre-COVID-19 to 2.31% COVID-19 which was statistically significant. When looking at the confirmed positive cases, there was an increase from 0.65% to 1.01% which was statistically significant (*p*=0.0052, Table 4)

## 4. Discussion

All the endodontic treatments that this study represented were performed using standard and advanced endodontic armamentarium, including microscopy, but did not utilize minimally invasive techniques such as contracted access, minimal preparations, or recent disinfection units, such as Gentlewave. The impact of these factors on the prevalence of VRF warrants further study.

Theoretically, VRFs start as small microcracks that grow over time [28]. They have been associated with postplacement and from heavy obturation forces. Maddalone et al. [3] found that when a post was placed, the prevalence of VRF increased to 16.2% compared to 1.2% for no post.

Excessive dentin removal during conventional root canal therapy may cause VRFs [5]. The etiology of the VRF is still unknown, yet minimally invasive root canal therapy is being recommended to minimize its prevalence. However, these changes in access and root canal preparation may lead to possible missed anatomy, incomplete disinfection, insufficient obturation, and increased the binding of instruments [29].

There was a difference in the prevalence between the UEP setting (overall 1.80%) and the PP setting (overall 2.62%). The UEP adopted digital radiography from 2011 which led to differences in the sample size and longer duration of data collection in the PP group which could explain the difference in prevalence between the two groups. Another reason for higher VRF prevalence in PP seemed to be higher number of retreatment cases being treated compared to an advanced program in dental school. Data from both the UEP and the PP show an increase in VRFs in all categories from pre-COVID-19 to COVID-19. This increase could be associated with the increase in overall stress from COVID-19. This increase in overall stress may have an influence in the masticatory stresses in the patients which could be a subject for another investigation. Our results showed that although the duration of the COVID-19 period was much shorter than the pre-COVID-19 period, prevalence of VRF was doubled during the former period compared to the latter. This is of interest as precautions against COVID-19 might have hampered regular dental treatments and follow-ups leading to deteriorated oral status.

Limitations of the study included that the prevalence was evaluated via treatment notes in Axium for UEP or treatment letters for PP. Both included searches of the “terms” to retrieve the count. Though the notes were sorted and analyzed for terms, there are still possibilities where certain notes may not have been included due to misspellings or variations of the terms. A prospective study design would overcome such limitations. The category “suspected” might have included situations with clinical and radiographic presentations similar to a VRF such as endo-perio cases with sinus tract through the PDL. However, patients' election to treat, extract, or monitor the case precluded the visual confirmation of the VRF. Hence, these cases were not considered to exhibit VRF. This might have affected the reported prevalence since for the purpose of this study, confirmed cases were only those in which the VRF was visible on the radiographs, after retraction of the gingiva or after raising a surgical flap while observation of VRF after extraction was not accepted as confirmation. This decision was based on the fact that the extraction procedure itself might induce VRF [2]. Our radiographic assessment was based on digital radiographs because only few cases had CBCT assessments. This might have reduced the detection rate of VRF in some cases. Nevertheless, scientific evidence supporting higher ability of CBCT to precisely detect VRF is still lacking [24].

For the UEP set, there was a total of 21,156 patients with a total number of 60,773 Axium records. These may have notes that addressed multiple teeth in one note which could deflate the prevalence. On the other hand, there may be multiple VRFs in a patient which could inflate the prevalence. Compared to the private practice, the number of patients matched up with the number of treatment letters. However, the treatment letters may also have had multiple teeth included in the letter.

Due to limitations of the study, radiographic confirmation for the UEP set was not available. If the note did not specify in detail the radiographic findings of a fracture, then it was not considered radiographically confirmed. Fortunately, the treatment letters from the PP provided images of clear digital periapical radiographs where the fractures could be seen leading to a more accurate data point.

It was also noticed when going through the data that there are some misunderstandings of what a VRF entails. There were crown-originating cracks and split teeth that were mislabeled as VRFs. Understanding the definition of a VRF is paramount to diagnosis and treatment planning.

The literature indicates that the best way to diagnose a VRF is to visualize it clinically [30]. The results of this study indicated that a large number of practitioners tend to suspect the VRF rather than confirm its presence. This study did not look at what percentage of those suspected went toward extraction or treatment which could be a future aim for investigation.

## 5. Conclusion

VRF was not a common finding from the obtained data. The prevalence of VRF in the UEP was 1.80% and 2.62% in PP. The combined prevalence for both settings was 2.01%. There was an increase in the prevalence of VRFs during COVID-19. Cracks or crown-originating fractures were difficult to diagnose and often confused with VRF. A VRF is a condemning finding. It is important to confirm that the VRF is present visually prior to recommending an extraction.

## Figures and Tables

**Table 1:** Prevalence of VRF in both clinical settings.

	University endodontics program		Private endodontics practice		Total (%)
	Total number	Prevalence (%)	Total number	Prevalence (%)	
All years	21,156	1.80	7,209	2.62	2.01
Pre-COVID-19	18,449	1.52	6,191	2.30	1.72
COVID-19	2,834	3.53	1,018	4.62	3.82

**Table 2:** University endodontics program: prevalence of suspected, confirmed positive, and confirmed negative.

	Total count			Prevalence			*p*-Value
	All years 21,156	Pre-COVID-19 18,449	COVID-19 2,834	All years (%)	Pre-COVID-19	COVID-19 (%)	
Suspected	197	143	54	0.93	0.78	1.91	**<0.0001**
Confirmed positive	147	119	28	0.69	0.65	0.99	**0.0202**
Radiographically	10	8	2	0.05	0.04	0.07	0.2676
Visually	52	45	7	0.25	0.24	0.25	0.4840
Surgically	85	66	19	0.40	0.36	0.67	**0.0071**
Confirmed negative	37	19	18	0.17	0.10	0.64	**<0.0001**
Total	381	281	100	1.80	1.52	3.53	**<0.0001**

Bold values signify the significant values (*p* < 0.05).

**Table 3:** Private endodontic practice: prevalence of suspected, confirmed positive, and confirmed negative.

	Total count			Prevalence			*p*-Value
	All years 7,209	Pre-COVID-19 6,191	COVID-19 1,018	All years (%)	Pre-COVID-19 (%)	COVID-19 (%)	
Suspected	135	100	35	1.87	1.62	3.44	**<0.0001**
Confirmed positive	52	41	11	0.72	0.66	1.08	0.0721
Radiographically	14	5	9	0.19	0.08	0.88	**<0.0001**
Visually	25	23	2	0.35	0.37	0.20	0.8106
Surgically	13	13	0	0.18	0.21	0.00	0.9279
Confirmed negative	2	1	1	0.03	0.02	0.10	0.0735
Total	189	142	47	2.62	2.29	4.62	**<0.0001**

Bold values signify the significant values (*p* < 0.05).

**Table 4:** Overall prevalence suspected and confirmed.

	Total Count			Prevalence (per patient)			*p*-Value
	All years	Pre-COVID-19	COVID-19	All years (%)	Pre-COVID-19 (%)	COVID-19 (%)	
Suspected	332	243	89	1.17	0.99	2.31	**<0.0001**
Confirmed positive	199	160	39	0.70	0.65	1.01	**0.0052**
Radiographically	24	13	11	0.08	0.05	0.29	**<0.0001**
Visually	77	68	9	0.27	0.28	0.23	0.6808
Surgically	98	79	19	0.35	0.32	0.49	**0.0446**
Confirmed negative	39	20	19	0.14	0.08	0.49	**<0.0001**
Total	570	423	147	2.01	1.72	3.82	**<0.0001**

Bold values signify the significant values (*p* < 0.05).

## Data Availability

The data used to support the findings of this study are available from the corresponding author upon request.
